# ZCURVE_V: a new self-training system for recognizing protein-coding genes in viral and phage genomes

**DOI:** 10.1186/1471-2105-7-9

**Published:** 2006-01-10

**Authors:** Feng-Biao Guo, Chun-Ting Zhang

**Affiliations:** 1 Department of Physics, Tianjin University, Tianjin 300072, China

## Abstract

**Background:**

It necessary to use highly accurate and statistics-based systems for viral and phage genome annotations. The GeneMark systems for gene-finding in virus and phage genomes suffer from some basic drawbacks. This paper puts forward an alternative approach for viral and phage gene-finding to improve the quality of annotations, particularly for newly sequenced genomes.

**Results:**

The new system ZCURVE_V has been run for 979 viral and 212 phage genomes, respectively, and satisfactory results are obtained. To have a fair comparison with the currently available software of similar function, GeneMark, a total of 30 viral genomes that have not been annotated by GeneMark are selected to be tested. Consequently, the average specificity of both systems is well matched, however the average sensitivity of ZCURVE_V for smaller viral genomes (< 100 kb), which constitute the main parts of viral genomes sequenced so far, is higher than that of GeneMark. Additionally, for the genome of *Amsacta moorei *entomopoxvirus, probably with the lowest genomic GC content among the sequenced organisms, the accuracy of ZCURVE_V is much better than that of GeneMark, because the later predicts hundreds of false-positive genes. ZCURVE_V is also used to analyze well-studied genomes, such as HIV-1, HBV and SARS-CoV. Accordingly, the performance of ZCURVE_V is generally better than that of GeneMark. Finally, ZCURVE_V may be downloaded and run locally, particularly facilitating its utilization, whereas GeneMark is not downloadable. Based on the above comparison, it is suggested that ZCURVE_V may serve as a preferred gene-finding tool for viral and phage genomes newly sequenced. However, it is also shown that the joint application of both systems, ZCURVE_V and GeneMark, leads to better gene-finding results. The system ZCURVE_V is freely available at: .

**Conclusion:**

ZCURVE_V may serve as a preferred gene-finding tool used for viral and phage genomes, especially for anonymous viral and phage genomes newly sequenced.

## Background

Developments of DNA sequencing technology have resulted in a rapid expansion of genome data. It becomes a challenging issue to explore the secrets of genomes and maximize the scientific knowledge gained from them. The first step in analyzing a completely or partially sequenced genome is to identify all its genes. Accurate gene recognition is relevant to many biological applications, for example, DNA microarray, knockout experiments and drug design. There exist some well-known computer systems for gene-finding in bacterial and archaeal genomes. These systems are either based on statistic analysis, such as GeneMarkS [1], Glimmer [2,3], and ZCURVE [4], or based on similarity alignment, such as CRITICA [5] and ORPHEUS [6]. Generally, satisfactory predicted results are obtained by using the above statistics-based software. On the contrary, genome annotation in newly sequenced viruses and phages is frequently based on similarity search methods such as BLAST [7]. Some species-specific genes are likely to be missed although high specificity is obtained by using similarity search methods. Evidence shows that an open reading frame (ORF) longer than a given length and not or slightly overlapping with any adjacent ORFs is likely to be a gene. However, simply assigning all such ORFs to genes usually generates over-predictions. Therefore, it is of necessity to use highly accurate and statistics-based systems for viral genome annotations. Unfortunately, currently there are very few satisfactory statistics-based viral gene-finding systems, except GeneMark gene-finding family [8,9]. However, GeneMark systems for gene-finding in virus and phage genomes suffer from some basic drawbacks. It is the aim of this paper to put forward an alternative approach for viral and phage gene-finding to improve the quality of annotations, particularly, for newly sequenced genomes.

The ZCURVE system for finding protein-coding genes in bacterial and archaeal genomes developed by our group has been used in 40 laboratories or institutes all over the world [4]. In a recent paper, ZCURVE and the other two well-known bacterial gene-finding systems, Glimmer and CRITICA, are combined into a metatool named YACOP [10]. By adapting similar algorithm of ZCURVE, a new system specific to coronavirus genomes, ZCURVE_CoV, has been developed subsequently [11]. The ZCURVE_CoV system results in highly consistent results with GenBank annotations for coronavirus genomes, especially for SARS-CoV genomes [11]. However, the above software cannot be simply used to identify protein-coding genes in other viral or phage genomes. Here, a self-training system, ZCURVE_V is presented to address the problem. Similar to ZCURVE [4] and ZCURVE_CoV [11], the present ZCURVE_V system is also based on the Z curve representation of DNA sequences [12]. Compared with the most widely used viral gene-finding system, GeneMark family [8,9], the algorithm of ZCURVE_V is much simpler, because only 33 recognition variables are needed. Therefore, ZCURVE_V is conceptually different from GeneMark. Compared with GeneMark, ZCURVE_V resulted in better predicted results for smaller viral genomes (< 100 kb). In addition, the performance of ZCURVE_V is generally better than that of GeneMark for genomes with particular features, such as amsacta moorei entomopoxvirus, probably with the lowest genomic GC content among all the organisms sequenced so far. Moreover, it is also shown that joint applications of ZCURVE_V and GeneMark lead to better gene-finding results for viral and phage genomes.

## Results and Discussions

### Indices to evaluate ZCURVE_V

The ZCURVE_V system has been run for 979 viral and 212 phage genome records, respectively. The default settings are adopted for all the options unless indicated otherwise. Evaluation of ZCURVE_V is based on the comparison between the gene-finding results and the RefSeq annotations for each genome. It should be noted that the RefSeq records are usually listed as provisional and have not themselves undergone extensive curation and literature cross-checking. However, to test and compare the performance of the presented algorithm we do need some criteria. Knowing that the RefSeq records are questionable, we chose to select those RefSeq data which possess the maximum reliability. For example, gene annotations in HIV, HBV and coronavirus are well known in the literature. Therefore, these three viruses are selected as samples to test and compare the algorithm. Other RefSeq records are selected similarly. Due to the inaccuracy of the RefSeq annotations currently available, the comparison between the performance of GeneMark and ZCURVE_V based on the RefSeq annotations should be deemed as preliminary. Future and more reliable comparison should be based on experimentally verified data, rather than RefSeq annotations. Two independent indices defined by formulas (1) and (2) are used to evaluate the performance of ZCURVE_V [13]

Sn=TPTP+FN,     1
 MathType@MTEF@5@5@+=feaafiart1ev1aaatCvAUfKttLearuWrP9MDH5MBPbIqV92AaeXatLxBI9gBaebbnrfifHhDYfgasaacH8akY=wiFfYdH8Gipec8Eeeu0xXdbba9frFj0=OqFfea0dXdd9vqai=hGuQ8kuc9pgc9s8qqaq=dirpe0xb9q8qiLsFr0=vr0=vr0dc8meaabaqaciaacaGaaeqabaqabeGadaaakeaacqWGtbWudaWgaaWcbaGaemOBa4gabeaakiabg2da9maalaaabaGaemivaqLaemiuaafabaGaemivaqLaemiuaaLaey4kaSIaemOrayKaemOta4eaaiabcYcaSiaaxMaacaWLjaacbeGae8xmaedaaa@3B71@

Sp=TPTP+FP,     2
 MathType@MTEF@5@5@+=feaafiart1ev1aaatCvAUfKttLearuWrP9MDH5MBPbIqV92AaeXatLxBI9gBaebbnrfifHhDYfgasaacH8akY=wiFfYdH8Gipec8Eeeu0xXdbba9frFj0=OqFfea0dXdd9vqai=hGuQ8kuc9pgc9s8qqaq=dirpe0xb9q8qiLsFr0=vr0=vr0dc8meaabaqaciaacaGaaeqabaqabeGadaaakeaacqWGtbWudaWgaaWcbaGaemiCaahabeaakiabg2da9maalaaabaGaemivaqLaemiuaafabaGaemivaqLaemiuaaLaey4kaSIaemOrayKaemiuaafaaiabcYcaSiaaxMaacaWLjaacbeGae8Nmaidaaa@3B7B@

where *TP*, *FP *and *FN *are the positively true, false positive and false negative predictions, respectively.

### Comparisons with GeneMark (I): viral genomes with different chromosome lengths

GeneMark gene-finding web server provides two alternative approaches for viral genome annotation, i.e., the online prediction using a heuristic approach (or using GeneMarks program for viral genomes longer than 100 kb) and the VIOLIN database [1,8,9]. Generally speaking, the results obtained by the latter are more accurate than those obtained by the former. To strictly evaluate the performance of ZCURVE_V, the predicted results deposited in the GeneMark VIOLIN database are employed unless they are not available. A total of 30 viral genomes not annotated by GeneMark were used for the comparison, whose names are listed as follows: canarypox virus (abbreviation name: CNPV, RefSeq AC: NC_005309), fowlpox virus (FPV, NC_002188), tupaia herpesvirus (THV, NC_002794), african swine fever virus (ASFV, NC_001659), myxoma virus (MYXV, NC_001132), shope fibroma virus (SFV, NC_001266), yaba-like disease virus (YLDV, NC_002642), orf virus (ORFV, NC_005336), bovine papular stomatitis virus (BPSV, NC_005337), *Autographa californica *nucleopolyhedrovirus (AcNPV, NC_001623), *Bombyx mori *nucleopolyhedrovirus (BmNPV, NC_001962), *Phthorimaea operculella *granulovirus (PhoGV, NC_004062), *Adoxophyes honmai *nucleopolyhedrovirus (AdhoNPV, NC_004690), lymphocystis disease virus 1 (LCDV-1, NC_005902), *Plutella xylostella *granulovirus (PxGV, NC_002593), *Adoxophyes orana *granulovirus (AdorGV, NC_005038), *Neodiprion lecontei *nucleopolyhedrovirus (NeleNPV, NC_005906), fowl adenovirus D (FAdV-9, NC_000899), porcine adenovirus C (PAdV-5, NC_002702), avian infectious bronchitis virus (IBV, NC_001451), citrus tristeza virus (CTV, NC_001661), simian hemorrhagic fever virus (SHFV, NC_003092), beet yellows virus (BYV, NC_001598), fer-de-lance virus (FDLV, NC_005084), equine arteritis virus (EAV, NC_002532), semliki forest virus (SFV, NC_003215), bean common mosaic necrosis virus (BCMV, NC_004047), garlic latent virus (GLV, NC_003557), figwort mosaic virus (FMV, NC_003554), and southern cowpea mosaic virus (SCMV, NC_001625), respectively. The predicted results for the 30 viral genomes are listed in Table 1, where the genomes are listed in the order in which the chromosome sequence length is descending. For the 15 viral genomes with the chromosome sequence length larger than 100 kb in Table 1, the average *S*_*n *_of ZCURVE_V and GeneMark is 95.9% and 92.5%, respectively, and the average *S*_*p *_of ZCURVE_V and GeneMark is 92.8% and 94.0%, respectively. For the 15 viral genomes with the chromosome sequence length less than 100 kb listed in Table 1, the average *S*_*n *_of ZCURVE_V and GeneMark is 95.6% and 80.0%, whereas the average *S*_*p *_of ZCURVE_V and GeneMark is 93.2% and 91.1%, respectively. As can be seen, both the average *S*_*n *_and *S*_*p *_of ZCURVE_V for the small viral genomes are similar with those for the large viral genomes, whereas the average *S*_*n *_of GeneMark for small viral genomes is much lower than that for large viral genomes. Note that viral and phage genomes shorter than 100 kb constitute the major part of viral and phage genomes sequenced so far. Over 90% of the 979 viral and 212 phage genomes analyzed here are shorter than 100 kb. If the average is performed over all the 30 genomes, *S*_*n *_and *S*_*p *_are 95.7% and 93.0% for ZCURVE_V, respectively, whereas *S*_*p *_and *S*_*p *_are 86.2% and 92.5% for GeneMark. In summary, *S*_*p *_of both systems is well matched, but *S*_*n *_of ZCURVE_V is much higher (about 9.5% higher) than that of GeneMark. Although Glimmer (2,3) were designed for gene-finding in bacterial genomes, for comparison, the gene-finding results by Glimmer 2.02 for all the 30 genomes are also listed in Table 1.

**Table 1:** The numbers of annotated and additional genes found by ZCURVE_V and GeneMark gene-finding family, respectively, for 30 viral genomes with different chromosome lengths ^a^

GenBank information				ZCURVE_V			GeneMark^b^			Glimmer ^d^		
Organisms	Sequence length (bp)	GC content	No. of annotated genes	No. of predicted genes	*S*_*n*_	*S*_*p*_	No. of predicted genes	*S*_*n*_	*S*_*p*_	No. of predicted genes	*S*_*n*_	*S*_*p*_

CNPV	359,853	30.37	328	342	99.4	95.3	327	97.9	98.2	351	99.1	92.6
FPV	288,539	30.89	261	282	95.4	88.3	257	93.5	94.9	307	95.0	80.8
THV	195,859	66.61	158	199	90.5	71.9	109	57.6	83.5	207	50.6	38.6
ASFV	170,101	38.95	151	164	96.0	88.4	148	93.4	95.3	185	95.4	77.8
MYXV	161,773	43.56	170	170	98.8	98.8	172	97.6	96.5	180	98.2	92.8
SFV	159,857	39.53	165	170	96.4	93.5	168	97.0	95.2	188	98.2	86.2
YLDV	144,575	27.00	152	156	99.3	96.8	155	98.7	96.8	165	98.7	90.9
ORFV	139,962	63.44	130	131	92.3	91.6	133	91.5	89.5	187	97.7	67.9
BPSV	134,431	64.50	131	144	96.9	88.2	135	93.9	91.1	150	86.3	75.3
AcNPV	133,894	40.70	155	155	97.4	97.4	152	94.8	96.7	174	96.2	86.2
BmNPV	128,413	40.40	143	139	97.2	100	139	95.1	97.8	157	95.8	87.3
PhopGV	119,217	35.7	130	132	96.2	94.7	130	93.1	93.1	168	96.9	75.0
AdhoNPV	113,220	35.64	125	121	92.0	95.0	125	94.4	94.4	143	95.2	83.2
LCDV-1	102,653	29.07	110	112	97.3	95.5	110	96.4	96.4	114	98.2	94.7
PxGV	100,999	40.69	120	116	93.3	96.6	123	92.5	90.2	131	94.2	86.3
AdorGV	99,657	34.49	119	121	97.5	95.9	116	94.1	96.6	137	95.8	83.2
NeleNPV	81,755	33.31	93	101	92.5	85.1	73	75.3	95.9	125	91.4	68.0
FAdV-9	45,063	53.78	29	48	100	60.4	35	96.6	80	60	100	48.3
PAdV-5	32,621	50.50	30	35	90.0	77.1	27	76.7	85.2	39	90.0	69.2
IBV	27,608	37.93	10	10	90.0	90.0	7	70	100	10	70.0	70.0
CTV	19,296	45.27	11	11	100	100	8	72.7	100	12	90.9	83.3
SHFV	15,717	50.11	11	11	100	100	8	54.5	75	--	--	--
BYV	15,480	46.03	8	8	100	100	7	87.5	100	11	100	72.7
FDLV	15,378	43.13	8	8	100	100	8	100	100	11	100	72.7
EAV	12,704	51.66	9	9	88.8	88.8	6	55.5	83.3	11	77.8	63.6
SFV	11,442	53.22	2	2	100	100	2	100	100	--	--	--
BCMV	9612	42.22	1	1	100	100	2	100	50	3	100	33.3
GLV	8363	43.86	6	6	100	100	4	66.7	100	7	50	42.9
FMV	7743	35.36	7	7	100	100	7	100	100	7	100	100
SCMV	4194	51.55	4	3	75	100	2	50	100	2	50	100
Average (upper 15)^c^	-	-	-	-	95.9 95.9	92.8	-	92.5	94.0	-	93.05	81.0
Average (lower 15)^c^	-	-	-	-	95.6	93.2	-	80.0	91.1	-	85.84	69.8
Average (30)^c^	-	-	-	-	95.7	93.0	-	86.2	92.5	-	89.70	75.8

^a ^The names of the viruses are listed in the descending order of their chromosome sequence lengths. The abbreviation names of viruses are used. See the text for the detail.

^b ^For the genomes of canarypox virus (CNPV), orf virus (ORFV), bovine papular stomatitis virus (BPSV) and neodiprion lecontei nucleopolyhedrovirus (NeleNPV), genes were predicted directly by GeneMarks program, whereas for the other 26 viral genomes the data deposited in the GeneMark VIOLIN database are used.

^c ^The values are averaged over the upper 15, lower 15 and all the 30 viral genomes, respectively.

^d ^Glimmer 2.02 predicted no genes for simian hemorrhagic fever virus (SHFV) and semliki forest virus (SFV) genomes.

### Comparisons with GeneMark (II): viral genomes with particular genomic features

Among the viruses curated by NCBI staff, two satellite viruses have genomic sequences shorter than 1000 bp, which are the cereal yellow dwarf virus-RPV satellite RNA (CYDV-RPV satRNA, NC_003533) and panicum mosaic satellite virus (satPaMV, NC_003847). Satellite maize white line mosaic virus (SV-MWLMV, NC_003631) and strawberry latent ringspot virus satellite RNA (SLRSV, NC_003848) have the sequence length a little bit longer than 1000 bp. As can be seen from Table 2, the gene-finding results of ZCURVE_V are more consistent with the RefSeq annotations than those of GeneMark for the four very small viral genomes.

**Table 2:** The numbers of annotated and additional genes found by ZCURVE_V and the GeneMark VIOLIN database, respectively, for the five genomes with particular features ^a^

GenBank information			ZCURVE_V		GeneMark^b^	
Organisms	Chromosome sequence length (bp)	No. of annotated genes	No. of annotated genes found	No. of additional genes predicted	No. of annotated genes found	No. of additional genes predicted

CYDV-RPV satRNA	322	0	0	0	0	0
SatPaMV^c^	826	2	2	0	1	0
SV-MWLMV	1168	1	1	0	0	1
SLRSV	1118	1	1	0	1	0
AmEPV	232,392	294	245	5	239	323

^a ^Of the five viral genomes, cereal yellow dwarf virus-RPV satellite RNA (CYDV-RPV satRNA), panicum mosaic satellite virus (satPaMV), satellite maize white line mosaic virus (SV-MWLMV) and strawberry latent ringspot virus satellite RNA (SLRSV) are less than or slightly larger than 1000 bp in length, whereas amsacta moorei entomopoxvirus (AmEPV) has probably the lowest GC content among the sequenced organisms (17.78%).

^b ^Data deposited in the GeneMark VIOLIN database are used.

^c ^For this genome, we adjusted the default settings, i.e., using the 'single-stranded virus' option.

The genome of *Amsacta moorei *entomopoxvirus (AmEPV, NC_002520) was sequenced in 2000 [14]. To our knowledge, it has the lowest genomic GC content among all the organisms completely sequenced so far, which is 17.78%. In the original annotation by the submitter of GenBank entries, all of the ORFs larger than 180 bp are predicted as possible protein-coding genes [14]. Such annotation method is very likely to generate over-annotation. The current RefSeq annotation curated by NCBI staff remains nearly the same compared with the original annotation, i.e., the genome contains 295 possible genes. After running ZCURVE_V, 245 out of the 295 annotated genes are found and the number of additionally predicted genes is 5. Among the 50 (295-245) genes not predicted by ZCURVE_V, only one gene has putative function and another two are similar to existing genes without functions in public databases, while the remaining 47 are only annotated as 'hypothetical proteins'. The result supports the notion that protein-coding genes are over-annotated in the amsacta moorei entomopoxvirus genome. The GeneMark VIOLIN database correctly predicts 239 annotated genes while the number of additionally predicted genes is as high as 323. It is obvious that most of these additional genes predicted by the GeneMark VIOLIN database are non-coding ORFs. Perhaps the severe over-prediction of the GeneMark VIOLIN database for the amsacta moorei entomopoxvirus genome is caused by its weak adaptability to genomes with particular features.

### Applying ZCURVE_V to HIV-1, HBV and SARS-CoV genomes

According to the report "AIDS Epidemic Update 2004" launched by WHO and UNAIDS: the total number of people living with the human immunodeficiency virus (HIV) increased in 2004 to reach its highest level ever: an estimated 39.4 million people are living with the virus [15]. The global AIDS epidemic killed 3.1 million people in the past year. In the current GenBank annotation for HIV-1 (GenBank AC: AF033819), 9 protein-coding genes are contained, in which 7 genes are single-exon genes without any intron. Genes *tat *and *rev *have one intron, respectively. When using default settings, ZCURVE_V and the GeneMark VIOLIN database predict 7 and 6 genes for the genome, respectively. The predicted results are listed in Table 3. As can be seen, both ZCURVE_V and the GeneMark VIOLIN database predict the 5 annotated single-exon genes *pol*, *gag*, *vpr*, *env *and *nef*. The single-exon gene *vif *is correctly predicted by ZCURVE_V, whereas the GeneMark VIOLIN database misses it. The single-exon gene *vpu *is correctly predicted by the GeneMark VIOLIN database, whereas ZCURVE_V misses it. In addition, ZCURVE_V correctly predicts the 5' end for the intron-contained gene *tat*. After adjusting the default settings, i.e., using the 'Keep Overlapping Genes' option, the gene *vpu *and one additional gene located at positions 7602–7694 bp are predicted by ZCURVE_V.

**Table 3:** Genes annotated and predicted by ZCURVE_V and the GeneMark VIOLIN database for human immunodeficiency virus 1 (HIV-1) ^a^

Genes annotated				Genes predicted by ZCURVE_V			Genes predicted by GeneMark		
Start	Stop	Length (aa)	Gene	Start	Stop	Length (aa)	Start	Stop	Length (aa)

336	1838	501	*gag*	336	1838	501	336	1838	501
1631	4642	1004	*Pol*	1904	4642	913	1904	4642	913
4587	5165	193	*Vif*	4587	5165	193			
5105	5341	79	*Vpr*	5105	5341	237	5105	5341	237
5377	7970	87	*Tat*	***5377***	***5595***	73			
5516	8199	117	*Rev*						
5608	5856	83	*Vpu*	**5608**	**5856**	**83**	5608	5856	83
5771	8341	857	*Env*	5771	8341	857	5771	8341	857
				**7602**	**7694**	**31**			
8343	8714	124	*Nef*	8343	8714	124	8343	8714	124

^a ^Bold denotes gene found by adapting the default settings of ZCURVE_V, i.e., keeping the overlapping genes. Bold and italic figures are associated with the gene, in which the 3' end is not consistent with annotated one, but is embedded within it.

Hepatitis B virus is another virus that severely threatens human health. Currently, GenBank annotation contains 4 single-exon genes for HBV (GenBank AC: X04615). Among the 4 genes, gene P is jointly composed by two fragments. When using default settings, ZCURVE_V and the GeneMark VIOLIN database predict 3 and 2 genes for the genome, respectively. The predicted results are listed in Table 4. As can be seen, both ZCURVE_V and the GeneMark VIOLIN database predict gene P. Gene C is correctly predicted by ZCURVE_V, but the GeneMark VIOLIN database misses it. Gene X is correctly predicted by GeneMark, but ZCURVE_V misses it. In addition, ZCURVE_V predicts one additional gene that is embedded within gene P. After adjusting the default settings, i.e., using the 'Keep Overlapping Genes' option, gene S and X are also correctly predicted by ZCURVE_V.

**Table 4:** Genes annotated and predicted by ZCURVE_V and the GeneMark VIOLIN database for hepatitis B virus (HBV) ^a^

Genes annotated				Genes predicted by ZCURVE_V			Genes predicted by GeneMark		
Start	Stop	Length (aa)	Gene	Start	Stop	Length (aa)	Start	Stop	Length (aa)

1	1623	541	P	421	1623	401	421	1623	401
155	835	227	S	**155**	**835**	**227**			
1374	1838	155	X	**1374**	**1838**	**155**	1374	1838	155
1901	2452	184	C	1814	2452	213			
2307	3215	303	P	***2446***	***2604***	***53***			

^a ^Bold denotes gene found by adapting the default settings of ZCURVE_V, i.e., keeping the overlapping genes. Bold and italic figures are associated with the gene that is embedded within the annotated gene.

SARS is a life-threatening disease that spread to may countries around the world in 2003 [16]. SARS is caused by a novel coronavirus, called SARS-coronavirus or SARS-CoV. SARS-CoVs belong to coronavirus and their genomes are single-stranded [17]. Among the 14 protein-coding genes annotated in SARS-CoV TOR2 genome (NC_004718), 12 genes are found by the ZCURVE_V system. The two genes missed by it are completely or nearly completely embedded within other genes and are very unlikely to encode proteins [11], while the GeneMark VIOLIN annotation misses 4 ones out of the 14 annotated genes [9].

In summary, the gene-finding performance of ZCURVE_V for the three well studied life-threatening viruses is generally better than that of GeneMark.

### New genes missed by both RefSeq annotations and GenBank annotations

Gene-finding programs may be used to find new protein-coding genes that have been missed from the public databases. Using ZCURVE_V, we find some new genes missed from both the RefSeq annotations and GenBank annotations, which have significant similarities with other genes deposited in the public databases, as in the cases of the genomes of bacteriophage VT2-Sa (NC_000902), ectocarpus siliculosus virus (NC_002687) and pseudomonas phage D3 (NC_002484). The detailed predicted results of ZCURVE_V for the three genomes are listed in the Appendix, see [18]. Now let us inspect a new gene located at positions c4872–c5093 of phage VT2-Sa genome coding for a putative protein with 72 amino acids. Using a BLASTP search against NR databases, a significant similarities (E-value for BLASTP = 6e-25, Identities = 100%) with gene (RefSeq AC: NP_308832) has been found, implying that the predicted gene codes for a C4-type zinc finger protein in Stx1 and Stx2 converting bacteriophage genomes. It should be noted that the GeneMark VIOLIN database also misses this gene. Another noticeable new gene is located at the positions 55,832 bp-56,248 bp in the direct strand of the pseudomonas phage D3 genome. The amino acid sequence of the protein encoded by this gene is found to have a significant similarity (E-value for BLASP = 8e-15, Identities = 58%) with the phage holin protein (RefSeq AC: NP_743718) found in the pseudomonas putida KT2440 genome. It also has a significant similarity (E-value = 4e-08, Identities = 52%) with the lysis protein (RefSeq AC: NP_892111) found in the genome of bacteriophage PY54. Because the two new genes have very significant similarities with function-known genes in public databases, they are likely to be functional genes missed in both GenBank and RefSeq annotations. According to our suggestions to NCBI staff, now they have been included in the current RefSeq annotations (RefSeq AC: YP_089649 and YP_138545, respectively).

### Relationship between functions of predicted genes and their VZ scores

Compared with GeneMark, a more convenient feature of ZCURVE_V is that the coding potential scores VZ are provided for all of the predicted genes. The predicted genes with higher VZ scores have higher possibility to encode proteins. Bacteriophage P4 genome (NC_001609) is studied here as an example. As is shown in Table 5, all the predicted genes with VZ scores lower than 0.30 have no putative functions, in other words, all the function-known genes have the VZ scores higher than 0.30. On the other hand, it is possible that false positive predictions are generally associated with lower VZ scores. Therefore, the use of ZCURVE_V may reduce experimental expenses when studying functions of predicted genes by excising false positive predicted genes, based on the associated coding potential scores VZ.

**Table 5:** The relationship between the values of VZ score and functions of predicted proteins for the bacteriophage P4

Genes annotated				Genes predicted by ZCURVE_V			
Start	Stop	Strand	Function	Start	Stop	Strand	VZ score

247	648	+	Hypothetical protein	247	648	+	0.162
651	1718	+	Hypothetical protein	651	1718	+	0.111
1746	2540	-	Hypothetical protein	1746	2540	-	0.071
2607	3926	-	Integrase	2607	3926	-	0.345
__	__	__	__	3954	4103	-	0.307
4096	4431	-	Hypothetical protein	4096	4431	-	0.159
4636	6969	-	DNA primase	4636	6969	-	0.5000
6984	7304	-	Hypothetical protein	6984	7304	-	0.434
7440	7895	-	Hypothetical protein	7440	7895	-	0.380
7888	8175	-	helper derepression protein	7888	8256	-	0.425
8168	8584	-	Putative CI repressor	8168	8812	-	0.377
8764	9030	-	Transcriptional regulator	8764	9030	-	0.408
__	__	__	__	8991	9173	-	0.264
9583	10317	+	Head size determination protein sid	9583	10317	+	0.426
10,314	10,814	+	Transactivation protein	10,314	10,814	+	0.353
10,888	11,460	+	Amber mutation-suppressing protein	108,88	11,460	+	0.419

### Preferred utilization of ZCURVE_V in the annotation of anonymous viral genomes

All the GeneMark family, the heuristic approach and the VIOLIN database for viral and phage gene-finding have some limitations. Heuristic approach [8] is a self-training method and no human intervention is required during the running process. However, the performance of heuristic approach is generally worse than that of the GeneMark VIOLIN database [9]. The GeneMark VIOLIN database provides just an up-to-date analysis of newly sequenced viral genomes and is not able to be used to analyze anonymous viral genomes. Similar to the heuristic approach of GeneMark family, the ZCURVE_V is also a self-training method and enables analyzing any anonymous viral and phage genomes without any human intervention. Because the executable version of the program ZCURVE_V may be downloaded and run locally, it will be used more conveniently. More specific options when running ZCURVE_V strengthen its power. The prediction of ZCURVE_V is more accurate than that of GeneMark for viral or phage genomes shorter than 1000 bp. Therefore, it is suggested that ZCURVE_V may serve as a preferred gene-finding tool for viral and phage genomes, especially for anonymous viral and phage genomes newly sequenced. However, we should point out the limitations of ZCURVE_V when predicting genes for viruses that use alternative coding schemes. This includes RNA editing, splicing, polyprotein processing, etc. Generally, like GeneMark, ZCURVE_V cannot deal with the above special cases.

### Joint applications of ZCURVE_V and GeneMark gene-finding family

Both GeneMark and ZCURVE_V are based on statistical characteristics of coding (non-coding) sequences. However the former is Markov-chain-based and mainly considers the local characteristics of DNA sequence, whereas the latter is the Z-curve-based and lays stress on global characteristics. Due to the difference of inherent algorithm, the predictions of ZCURVE_V and GeneMark are different, although most of the predicted genes are identical. Higher accuracy may be obtained by combining them, in which genes predicted by either ZCURVE_V system or the GeneMark VIOLIN database are finally predicted as genes. Clover yellow mosaic virus (CLYMV, NC_001753), lymphocystis disease virus 1 (LCDV-1, NC_001824), ....transmissible gastroenteritis virus (TGEV, NC_002306) and yaba-like disease virus (YLDV, NC_002642) are chosen to demonstrate the effectiveness of joint applications of both systems. The results are listed in Table 6. As can be seen, the number of genes missed by the ZCURVE_V program decreases significantly although the number of additional predicted genes increases. Currently, it becomes a hotspot to develop an integrated genome annotation platform by joint applications of two or more systems based on different statistic analysis principles [10,19]. Similarly, joint applications of two or more viral gene-finding programs are also of necessity and feasibility. The programs of ZCURVE_V, GeneMark and others may all be jointed together to reach more accurate results. One referee of the manuscript points out that combining the use of prediction programs based on statistical measures such as ZCURVE_V with detection of functional motifs, sequence similarity, conservation of orthologs, presence of regulatory signals, etc., would be useful. Sequence similarity and conservation of orthologs methods may effectively reduce false positive predictions. Anyway, no one program can be used in isolation for making accurate predictions of the gene complement of any viral genome. Therefore use of multiple programs is always warranted. However, no concrete approach is provided to joint different information into a unified tool to reach the maximum accuracy. It seems that this is a topic of further study, not being included into the present paper.

**Table 6:** Joint applications of ZCURVE_V and GeneMark for the four viral genomes^a^

Organisms		CLYVV	LCDV-1	TGEV	YLDV
Annotated genes		5	110	9	152

ZCURVE_V	Annotated genes found	4	107	8	151
	Additional genes found	0	5	0	5
GeneMark VIOLIN	Annotated genes found	4	106	8	150
	Additional genes found	0	4	0	5
Joint	Annotated genes found	5	108	9	152
	Additional genes found	0	8	0	9

^a ^They are clover yellow mosaic virus (CLYMV), lymphocystis disease virus 1 (LCDV-1), ....transmissible gastroenteritis virus (TGEV) and yaba-like disease virus (YLDV) genomes, respectively.

## Conclusion

A new self-training system, ZCURVE_V, for finding genes in viral and phage genomes has been proposed. The new system ZCURVE_V has been run for 979 viral and 212 phage genomes, respectively, and satisfactory results are obtained. To have a fair comparison with the currently available software of similar function, GeneMark, a total of 30 viral genomes that have not been annotated by GeneMark are selected to be tested. Consequently, the average specificity of both systems is well matched, however, the average sensitivity of ZCURVE_V for smaller viral genomes (< 100 kb), which constitute the main parts of viral genomes sequenced so far, is higher than that of GeneMark. Additionally, for the genome of amsacta moorei entomopoxvirus, probably with the lowest genomic GC content among the sequenced organisms, the accuracy of ZCURVE_V is much better than that of GeneMark, because the later predicts hundreds of false-positive genes. ZCURVE_V is also used to analyze some well studied genomes, such as HIV-1, HBV and SARS-CoV. Accordingly, the performance of ZCURVE_V is generally better than that of GeneMark. Finally, GeneMark is not downloadable, whereas ZCURVE_V may be downloaded and run locally, particularly facilitating its utilization. Based on the above merits, it is suggested that ZCURVE_V may serve as a preferred gene-finding tool for viral and phage genomes newly sequenced. However, it is also shown that joint applications of both systems, ZCURVE_V and GeneMark, lead to better gene-finding results. The system ZCURVE_V is freely available at: .

## Methods

A total of 979 viral and 212 phage genome records were downloaded from GenBank release 141.0 [20]. Each record corresponds to a genome or a genomic segment. The corresponding RefSeq annotations for these genomes were downloaded before July 20, 2004 [21]. For all of the viral and phage genomes, the predicted results of the GeneMark VIOLIN database [22] were also downloaded before July 20, 2004.

The present gene-finding method consists of the four steps:

### (1) Extracting the seed ORF for the analyzed genome

In the present algorithm, only one seed ORF is required for a viral genome. This seed ORF is selected using a simple approach. It is found that an ORF with the largest length among all others in a genome is very likely to be a protein-coding gene. This ORF is called the 'Maximum ORF' in this paper. After carefully investigating over 100 viral genomes that have annotated genes, the deduction that the 'Maximum ORF' is a gene is valid accurately. For the two very small viral genomes, cereal yellow dwarf virus -RPV satellite RNA (NC_003533) and arabis mosaic virus small satellite RNA (NC_001546), there are no genes at al, indicating that the seed ORF so obtained is meaningless for these two genomes. If the 'Maximum ORF' is larger than 400 bp, it is directly regarded as a seed ORF (gene). However, if the 'Maximum ORF' is less than 400 bp, it is regarded as a seed ORF only if the base composition at the second codon position meets the following equation: *G*_2 _< (*A*_2 _+ *C*_2 _+ *T*_2_)/3 + 0.1, where *A*_2_, *C*_2_, *G*_2 _and *T*_2 _are the occurrence frequencies of bases at the second position of an ORF. This equation approximately reflects the fact that bases at the second codon position lack guanine to some degree [23]. If a seed ORF is found, then it will be used as a training sample to calculate the related parameters. Otherwise, if there is no seed ORF found, it means that the analyzed viral genome contains no functional genes.

### (2) Training the parameter used to describe the coding potential

The methodology adopted here is based on the Z curve [12], which is another representation of DNA sequence. Here the algorithm is presented briefly as follows. The frequencies of bases A, C, G and T occurring in an ORF or a fragment of DNA sequence with bases at positions 1, 4, 7, ...; 2, 5, 8, ..., and 3, 6, 9, ..., are denoted by *a*_1_, *c*_1_, *g*_1_, *t*_1_, *a*_2_, *c*_2_, *g*_2_, *t*_2_, *a*_3_, *c*_3_, *g*_3_, *t*_3 _respectively. They are actually the frequencies of bases at the 1^st^, 2^nd ^and 3^rd ^codon positions. Based on the Z curve (12), *a*_*i*_, *c*_*i*_, *g*_*i*_, *t*_*i *_are mapped onto a point P_i _in a 3-dimensinal space V_i_, *i *= 1, 2, 3. The coordinates of P_i_, denoted by *x*_*i*_, *y*_*i*_, *z*_*i*_, are determined by the Z-transform of DNA sequence [12].

{xi=(ai+gi)−(ci+ti),yi=(ai+ci)−(gi+ti),xi,yi,zi∈[−1,1],zi=(ai+ti)−(gi+ci).i=1,2,3.     3
 MathType@MTEF@5@5@+=feaafiart1ev1aaatCvAUfKttLearuWrP9MDH5MBPbIqV92AaeXatLxBI9gBaebbnrfifHhDYfgasaacH8akY=wiFfYdH8Gipec8Eeeu0xXdbba9frFj0=OqFfea0dXdd9vqai=hGuQ8kuc9pgc9s8qqaq=dirpe0xb9q8qiLsFr0=vr0=vr0dc8meaabaqaciaacaGaaeqabaqabeGadaaakeaadaGabaqaauaabaqadqaaaaqaaiabdIha4naaBaaaleaacqWGPbqAaeqaaOGaeyypa0JaeiikaGIaemyyae2aaSbaaSqaaiabdMgaPbqabaGccqGHRaWkcqWGNbWzdaWgaaWcbaGaemyAaKgabeaakiabcMcaPiabgkHiTiabcIcaOiabdogaJnaaBaaaleaacqWGPbqAaeqaaOGaey4kaSIaemiDaq3aaSbaaSqaaiabdMgaPbqabaGccqGGPaqkcqGGSaalaeaaaeaaaeaaaeaacqWG5bqEdaWgaaWcbaGaemyAaKgabeaakiabg2da9iabcIcaOiabdggaHnaaBaaaleaacqWGPbqAaeqaaOGaey4kaSIaem4yam2aaSbaaSqaaiabdMgaPbqabaGccqGGPaqkcqGHsislcqGGOaakcqWGNbWzdaWgaaWcbaGaemyAaKgabeaakiabgUcaRiabdsha0naaBaaaleaacqWGPbqAaeqaaOGaeiykaKIaeiilaWcabaGaemiEaG3aaSbaaSqaaiabdMgaPbqabaGccqGGSaalcqWG5bqEdaWgaaWcbaGaemyAaKgabeaakiabcYcaSaqaaiabdQha6naaBaaaleaacqWGPbqAaeqaaOGaeyicI4Saei4waSLaeyOeI0IaeGymaeJaeiilaWIaeGymaeJaeiyxa0LaeiilaWcabaaabaGaemOEaO3aaSbaaSqaaiabdMgaPbqabaGccqGH9aqpcqGGOaakcqWGHbqydaWgaaWcbaGaemyAaKgabeaakiabgUcaRiabdsha0naaBaaaleaacqWGPbqAaeqaaOGaeiykaKIaeyOeI0IaeiikaGIaem4zaC2aaSbaaSqaaiabdMgaPbqabaGccqGHRaWkcqWGJbWydaWgaaWcbaGaemyAaKgabeaakiabcMcaPiabc6caUaqaaaqaaaqaaaaacqWGPbqAcqGH9aqpcqaIXaqmcqGGSaalcqaIYaGmcqGGSaalcqaIZaWmcqGGUaGlaiaawUhaaiaaxMaacaWLjaacbeGae83mamdaaa@8F74@

The Z-transform of DNA sequence transforms the four frequencies of DNA bases into the coordinates of a point in a 3-dimensional space. In addition to the frequencies of codon-position-dependent single nucleotides, we need to consider the frequencies of phase-specific dinucleotides. Let the frequencies of the 16 dinucleotides AA, AC, ..., and TT occurring at the codon positions1-2 and 2–3 of an ORF or a fragment of DNA sequence be denoted by *p*_12_(AA), *p*_12_(AC), ...,*p*_12_(TT); *p*_12_(AA), *p*_12_(AC), ... and *p*_12_(TT) respectively. Using the Z-transform [12], we find

{xkX=(pk(XA)+pk(XG))−(pk(XC)+pk(XT)),ykX=(pk(XA)+pk(XC))−(pk(XG)+pk(XT)),X=A,C,G,T,k=12,23,zkX=(pk(XA)+pk(XT))−(pk(XG)+pk(XC)).     4
 MathType@MTEF@5@5@+=feaafiart1ev1aaatCvAUfKttLearuWrP9MDH5MBPbIqV92AaeXatLxBI9gBaebbnrfifHhDYfgasaacH8akY=wiFfYdH8Gipec8Eeeu0xXdbba9frFj0=OqFfea0dXdd9vqai=hGuQ8kuc9pgc9s8qqaq=dirpe0xb9q8qiLsFr0=vr0=vr0dc8meaabaqaciaacaGaaeqabaqabeGadaaakeaadaGabaqaauaabaqadmaaaeaacqWG4baEdaqhaaWcbaGaem4AaSgabaGaemiwaGfaaOGaeyypa0JaeiikaGIaemiCaa3aaSbaaSqaaiabdUgaRbqabaGccqGGOaakcqqGybawcqqGbbqqcqGGPaqkcqGHRaWkcqWGWbaCdaWgaaWcbaGaem4AaSgabeaakiabcIcaOiabbIfayjabbEeahjabcMcaPiabcMcaPiabgkHiTiabcIcaOiabdchaWnaaBaaaleaacqWGRbWAaeqaaOGaeiikaGIaeeiwaGLaee4qamKaeiykaKIaey4kaSIaemiCaa3aaSbaaSqaaiabdUgaRbqabaGccqGGOaakcqqGybawcqqGubavcqGGPaqkcqGGPaqkcqGGSaalaeaaaeaaaeaacqWG5bqEdaqhaaWcbaGaem4AaSgabaGaemiwaGfaaOGaeyypa0JaeiikaGIaemiCaa3aaSbaaSqaaiabdUgaRbqabaGccqGGOaakcqqGybawcqqGbbqqcqGGPaqkcqGHRaWkcqWGWbaCdaWgaaWcbaGaem4AaSgabeaakiabcIcaOiabbIfayjabboeadjabcMcaPiabcMcaPiabgkHiTiabcIcaOiabdchaWnaaBaaaleaacqWGRbWAaeqaaOGaeiikaGIaeeiwaGLaee4raCKaeiykaKIaey4kaSIaemiCaa3aaSbaaSqaaiabdUgaRbqabaGccqGGOaakcqqGybawcqqGubavcqGGPaqkcqGGPaqkcqGGSaalaeaacqqGybawcqGH9aqpcqqGbbqqcqGGSaalcqqGdbWqcqGGSaalcqqGhbWrcqGGSaalcqqGubavcqGGSaalaeaacqWGRbWAcqGH9aqpcqaIXaqmcqaIYaGmcqGGSaalcqaIYaGmcqaIZaWmcqGGSaalaeaacqWG6bGEdaqhaaWcbaGaem4AaSgabaGaemiwaGfaaOGaeyypa0JaeiikaGIaemiCaa3aaSbaaSqaaiabdUgaRbqabaGccqGGOaakcqqGybawcqqGbbqqcqGGPaqkcqGHRaWkcqWGWbaCdaWgaaWcbaGaem4AaSgabeaakiabcIcaOiabbIfayjabbsfaujabcMcaPiabcMcaPiabgkHiTiabcIcaOiabdchaWnaaBaaaleaacqWGRbWAaeqaaOGaeiikaGIaeeiwaGLaee4raCKaeiykaKIaey4kaSIaemiCaa3aaSbaaSqaaiabdUgaRbqabaGccqGGOaakcqqGybawcqqGdbWqcqGGPaqkcqGGPaqkcqGGUaGlaeaaaeaaaaaacaGL7baacaWLjaGaaCzcaGqabiab=rda0aaa@BAD0@

where xkX
 MathType@MTEF@5@5@+=feaafiart1ev1aaatCvAUfKttLearuWrP9MDH5MBPbIqV92AaeXatLxBI9gBaebbnrfifHhDYfgasaacH8akY=wiFfYdH8Gipec8Eeeu0xXdbba9frFj0=OqFfea0dXdd9vqai=hGuQ8kuc9pgc9s8qqaq=dirpe0xb9q8qiLsFr0=vr0=vr0dc8meaabaqaciaacaGaaeqabaqabeGadaaakeaacqWG4baEdaqhaaWcbaGaem4AaSgabaGaemiwaGfaaaaa@30EA@, ykX
 MathType@MTEF@5@5@+=feaafiart1ev1aaatCvAUfKttLearuWrP9MDH5MBPbIqV92AaeXatLxBI9gBaebbnrfifHhDYfgasaacH8akY=wiFfYdH8Gipec8Eeeu0xXdbba9frFj0=OqFfea0dXdd9vqai=hGuQ8kuc9pgc9s8qqaq=dirpe0xb9q8qiLsFr0=vr0=vr0dc8meaabaqaciaacaGaaeqabaqabeGadaaakeaacqWG5bqEdaqhaaWcbaGaem4AaSgabaGaemiwaGfaaaaa@30EC@ and zkX
 MathType@MTEF@5@5@+=feaafiart1ev1aaatCvAUfKttLearuWrP9MDH5MBPbIqV92AaeXatLxBI9gBaebbnrfifHhDYfgasaacH8akY=wiFfYdH8Gipec8Eeeu0xXdbba9frFj0=OqFfea0dXdd9vqai=hGuQ8kuc9pgc9s8qqaq=dirpe0xb9q8qiLsFr0=vr0=vr0dc8meaabaqaciaacaGaaeqabaqabeGadaaakeaacqWG6bGEdaqhaaWcbaGaem4AaSgabaGaemiwaGfaaaaa@30EE@ are the coordinates, X = A, C, G, T and *k *= 12, 23. Let the 3-dimensional space VkX
 MathType@MTEF@5@5@+=feaafiart1ev1aaatCvAUfKttLearuWrP9MDH5MBPbIqV92AaeXatLxBI9gBaebbnrfifHhDYfgasaacH8akY=wiFfYdH8Gipec8Eeeu0xXdbba9frFj0=OqFfea0dXdd9vqai=hGuQ8kuc9pgc9s8qqaq=dirpe0xb9q8qiLsFr0=vr0=vr0dc8meaabaqaciaacaGaaeqabaqabeGadaaakeaacqqGwbGvdaqhaaWcbaGaee4AaSgabaGaeeiwaGfaaaaa@30A0@ be spanned by xkX
 MathType@MTEF@5@5@+=feaafiart1ev1aaatCvAUfKttLearuWrP9MDH5MBPbIqV92AaeXatLxBI9gBaebbnrfifHhDYfgasaacH8akY=wiFfYdH8Gipec8Eeeu0xXdbba9frFj0=OqFfea0dXdd9vqai=hGuQ8kuc9pgc9s8qqaq=dirpe0xb9q8qiLsFr0=vr0=vr0dc8meaabaqaciaacaGaaeqabaqabeGadaaakeaacqWG4baEdaqhaaWcbaGaem4AaSgabaGaemiwaGfaaaaa@30EA@, ykX
 MathType@MTEF@5@5@+=feaafiart1ev1aaatCvAUfKttLearuWrP9MDH5MBPbIqV92AaeXatLxBI9gBaebbnrfifHhDYfgasaacH8akY=wiFfYdH8Gipec8Eeeu0xXdbba9frFj0=OqFfea0dXdd9vqai=hGuQ8kuc9pgc9s8qqaq=dirpe0xb9q8qiLsFr0=vr0=vr0dc8meaabaqaciaacaGaaeqabaqabeGadaaakeaacqWG5bqEdaqhaaWcbaGaem4AaSgabaGaemiwaGfaaaaa@30EC@ and zkX
 MathType@MTEF@5@5@+=feaafiart1ev1aaatCvAUfKttLearuWrP9MDH5MBPbIqV92AaeXatLxBI9gBaebbnrfifHhDYfgasaacH8akY=wiFfYdH8Gipec8Eeeu0xXdbba9frFj0=OqFfea0dXdd9vqai=hGuQ8kuc9pgc9s8qqaq=dirpe0xb9q8qiLsFr0=vr0=vr0dc8meaabaqaciaacaGaaeqabaqabeGadaaakeaacqWG6bGEdaqhaaWcbaGaem4AaSgabaGaemiwaGfaaaaa@30EE@. The direct-sum of the subspaces V_1_, V_2_, V_3_, V12A
 MathType@MTEF@5@5@+=feaafiart1ev1aaatCvAUfKttLearuWrP9MDH5MBPbIqV92AaeXatLxBI9gBaebbnrfifHhDYfgasaacH8akY=wiFfYdH8Gipec8Eeeu0xXdbba9frFj0=OqFfea0dXdd9vqai=hGuQ8kuc9pgc9s8qqaq=dirpe0xb9q8qiLsFr0=vr0=vr0dc8meaabaqaciaacaGaaeqabaqabeGadaaakeaacqqGwbGvdaqhaaWcbaGaeeymaeJaeeOmaidabaGaeeyqaeeaaaaa@30E9@, V12C
 MathType@MTEF@5@5@+=feaafiart1ev1aaatCvAUfKttLearuWrP9MDH5MBPbIqV92AaeXatLxBI9gBaebbnrfifHhDYfgasaacH8akY=wiFfYdH8Gipec8Eeeu0xXdbba9frFj0=OqFfea0dXdd9vqai=hGuQ8kuc9pgc9s8qqaq=dirpe0xb9q8qiLsFr0=vr0=vr0dc8meaabaqaciaacaGaaeqabaqabeGadaaakeaacqqGwbGvdaqhaaWcbaGaeeymaeJaeeOmaidabaGaee4qameaaaaa@30ED@, V12G
 MathType@MTEF@5@5@+=feaafiart1ev1aaatCvAUfKttLearuWrP9MDH5MBPbIqV92AaeXatLxBI9gBaebbnrfifHhDYfgasaacH8akY=wiFfYdH8Gipec8Eeeu0xXdbba9frFj0=OqFfea0dXdd9vqai=hGuQ8kuc9pgc9s8qqaq=dirpe0xb9q8qiLsFr0=vr0=vr0dc8meaabaqaciaacaGaaeqabaqabeGadaaakeaacqqGwbGvdaqhaaWcbaGaeeymaeJaeeOmaidabaGaee4raCeaaaaa@30F5@, V12T
 MathType@MTEF@5@5@+=feaafiart1ev1aaatCvAUfKttLearuWrP9MDH5MBPbIqV92AaeXatLxBI9gBaebbnrfifHhDYfgasaacH8akY=wiFfYdH8Gipec8Eeeu0xXdbba9frFj0=OqFfea0dXdd9vqai=hGuQ8kuc9pgc9s8qqaq=dirpe0xb9q8qiLsFr0=vr0=vr0dc8meaabaqaciaacaGaaeqabaqabeGadaaakeaacqqGwbGvdaqhaaWcbaGaeeymaeJaeeOmaidabaGaeeivaqfaaaaa@310F@, V23A
 MathType@MTEF@5@5@+=feaafiart1ev1aaatCvAUfKttLearuWrP9MDH5MBPbIqV92AaeXatLxBI9gBaebbnrfifHhDYfgasaacH8akY=wiFfYdH8Gipec8Eeeu0xXdbba9frFj0=OqFfea0dXdd9vqai=hGuQ8kuc9pgc9s8qqaq=dirpe0xb9q8qiLsFr0=vr0=vr0dc8meaabaqaciaacaGaaeqabaqabeGadaaakeaacqqGwbGvdaqhaaWcbaGaeeOmaiJaee4mamdabaGaeeyqaeeaaaaa@30ED@, V23C
 MathType@MTEF@5@5@+=feaafiart1ev1aaatCvAUfKttLearuWrP9MDH5MBPbIqV92AaeXatLxBI9gBaebbnrfifHhDYfgasaacH8akY=wiFfYdH8Gipec8Eeeu0xXdbba9frFj0=OqFfea0dXdd9vqai=hGuQ8kuc9pgc9s8qqaq=dirpe0xb9q8qiLsFr0=vr0=vr0dc8meaabaqaciaacaGaaeqabaqabeGadaaakeaacqqGwbGvdaqhaaWcbaGaeeOmaiJaee4mamdabaGaee4qameaaaaa@30F1@, V23G
 MathType@MTEF@5@5@+=feaafiart1ev1aaatCvAUfKttLearuWrP9MDH5MBPbIqV92AaeXatLxBI9gBaebbnrfifHhDYfgasaacH8akY=wiFfYdH8Gipec8Eeeu0xXdbba9frFj0=OqFfea0dXdd9vqai=hGuQ8kuc9pgc9s8qqaq=dirpe0xb9q8qiLsFr0=vr0=vr0dc8meaabaqaciaacaGaaeqabaqabeGadaaakeaacqqGwbGvdaqhaaWcbaGaeeOmaiJaee4mamdabaGaee4raCeaaaaa@30F9@ and V23T
 MathType@MTEF@5@5@+=feaafiart1ev1aaatCvAUfKttLearuWrP9MDH5MBPbIqV92AaeXatLxBI9gBaebbnrfifHhDYfgasaacH8akY=wiFfYdH8Gipec8Eeeu0xXdbba9frFj0=OqFfea0dXdd9vqai=hGuQ8kuc9pgc9s8qqaq=dirpe0xb9q8qiLsFr0=vr0=vr0dc8meaabaqaciaacaGaaeqabaqabeGadaaakeaacqqGwbGvdaqhaaWcbaGaeeOmaiJaee4mamdabaGaeeivaqfaaaaa@3113@ is denoted by a 33-dimensional space V, *i.e*., V = V_1 _⊕ V_2 _⊕ V_3 _⊕ V12A
 MathType@MTEF@5@5@+=feaafiart1ev1aaatCvAUfKttLearuWrP9MDH5MBPbIqV92AaeXatLxBI9gBaebbnrfifHhDYfgasaacH8akY=wiFfYdH8Gipec8Eeeu0xXdbba9frFj0=OqFfea0dXdd9vqai=hGuQ8kuc9pgc9s8qqaq=dirpe0xb9q8qiLsFr0=vr0=vr0dc8meaabaqaciaacaGaaeqabaqabeGadaaakeaacqqGwbGvdaqhaaWcbaGaeeymaeJaeeOmaidabaGaeeyqaeeaaaaa@30E9@ ⊕ ..... ⊕ V23T
 MathType@MTEF@5@5@+=feaafiart1ev1aaatCvAUfKttLearuWrP9MDH5MBPbIqV92AaeXatLxBI9gBaebbnrfifHhDYfgasaacH8akY=wiFfYdH8Gipec8Eeeu0xXdbba9frFj0=OqFfea0dXdd9vqai=hGuQ8kuc9pgc9s8qqaq=dirpe0xb9q8qiLsFr0=vr0=vr0dc8meaabaqaciaacaGaaeqabaqabeGadaaakeaacqqGwbGvdaqhaaWcbaGaeeOmaiJaee4mamdabaGaeeivaqfaaaaa@3113@, where the symbol ⊕ denotes the direct-sum of two subspaces. The 33 components of the space V, i.e., *u*_1_, *u*_2_, *..., u*_33_, are defined as follows

{u1=x1,u2=y1,u3=z1,u4=x2,u5=y2,u6=z2,u7=x3,u8=y3,u9=z3.     5
 MathType@MTEF@5@5@+=feaafiart1ev1aaatCvAUfKttLearuWrP9MDH5MBPbIqV92AaeXatLxBI9gBaebbnrfifHhDYfgasaacH8akY=wiFfYdH8Gipec8Eeeu0xXdbba9frFj0=OqFfea0dXdd9vqai=hGuQ8kuc9pgc9s8qqaq=dirpe0xb9q8qiLsFr0=vr0=vr0dc8meaabaqaciaacaGaaeqabaqabeGadaaakeaadaGabaqaauaabaqadmaaaeaacqWG1bqDdaWgaaWcbaGaeGymaedabeaakiabg2da9iabdIha4naaBaaaleaacqaIXaqmaeqaaOGaeiilaWcabaGaemyDau3aaSbaaSqaaiabikdaYaqabaGccqGH9aqpcqWG5bqEdaWgaaWcbaGaeGymaedabeaakiabcYcaSaqaaiabdwha1naaBaaaleaacqaIZaWmaeqaaOGaeyypa0JaemOEaO3aaSbaaSqaaiabigdaXaqabaGccqGGSaalaeaacqWG1bqDdaWgaaWcbaGaeGinaqdabeaakiabg2da9iabdIha4naaBaaaleaacqaIYaGmaeqaaOGaeiilaWcabaGaemyDau3aaSbaaSqaaiabiwda1aqabaGccqGH9aqpcqWG5bqEdaWgaaWcbaGaeGOmaidabeaakiabcYcaSaqaaiabdwha1naaBaaaleaacqaI2aGnaeqaaOGaeyypa0JaemOEaO3aaSbaaSqaaiabikdaYaqabaGccqGGSaalaeaacqWG1bqDdaWgaaWcbaGaeG4naCdabeaakiabg2da9iabdIha4naaBaaaleaacqaIZaWmaeqaaOGaeiilaWcabaGaemyDau3aaSbaaSqaaiabiIda4aqabaGccqGH9aqpcqWG5bqEdaWgaaWcbaGaeG4mamdabeaakiabcYcaSaqaaiabdwha1naaBaaaleaacqaI5aqoaeqaaOGaeyypa0JaemOEaO3aaSbaaSqaaiabiodaZaqabaGccqGGUaGlaaaacaGL7baacaWLjaGaaCzcaGqabiab=vda1aaa@7097@

{u10=x12A,u11=y12A,u12=z12A,u13=x12C,u14=y12C,u15=z12C,u16=x12G,u17=y12G,u18=z12G,u19=x12T,u20=y12T,u21=z12T,     5'
 MathType@MTEF@5@5@+=feaafiart1ev1aaatCvAUfKttLearuWrP9MDH5MBPbIqV92AaeXatLxBI9gBaebbnrfifHhDYfgasaacH8akY=wiFfYdH8Gipec8Eeeu0xXdbba9frFj0=OqFfea0dXdd9vqai=hGuQ8kuc9pgc9s8qqaq=dirpe0xb9q8qiLsFr0=vr0=vr0dc8meaabaqaciaacaGaaeqabaqabeGadaaakeaadaGabaqaauaabaqaemaaaaqaaiabdwha1naaBaaaleaacqaIXaqmcqaIWaamaeqaaOGaeyypa0JaemiEaG3aa0baaSqaaiabigdaXiabikdaYaqaaiabdgeabbaakiabcYcaSaqaaiabdwha1naaBaaaleaacqaIXaqmcqaIXaqmaeqaaOGaeyypa0JaemyEaK3aa0baaSqaaiabigdaXiabikdaYaqaaiabdgeabbaakiabcYcaSaqaaiabdwha1naaBaaaleaacqaIXaqmcqaIYaGmaeqaaOGaeyypa0JaemOEaO3aa0baaSqaaiabigdaXiabikdaYaqaaiabdgeabbaakiabcYcaSaqaaiabdwha1naaBaaaleaacqaIXaqmcqaIZaWmaeqaaOGaeyypa0JaemiEaG3aa0baaSqaaiabigdaXiabikdaYaqaaiabdoeadbaakiabcYcaSaqaaiabdwha1naaBaaaleaacqaIXaqmcqaI0aanaeqaaOGaeyypa0JaemyEaK3aa0baaSqaaiabigdaXiabikdaYaqaaiabdoeadbaakiabcYcaSaqaaiabdwha1naaBaaaleaacqaIXaqmcqaI1aqnaeqaaOGaeyypa0JaemOEaO3aa0baaSqaaiabigdaXiabikdaYaqaaiabdoeadbaakiabcYcaSaqaaiabdwha1naaBaaaleaacqaIXaqmcqaI2aGnaeqaaOGaeyypa0JaemiEaG3aa0baaSqaaiabigdaXiabikdaYaqaaiabdEeahbaakiabcYcaSaqaaiabdwha1naaBaaaleaacqaIXaqmcqaI3aWnaeqaaOGaeyypa0JaemyEaK3aa0baaSqaaiabigdaXiabikdaYaqaaiabdEeahbaakiabcYcaSaqaaiabdwha1naaBaaaleaacqaIXaqmcqaI4aaoaeqaaOGaeyypa0JaemOEaO3aa0baaSqaaiabigdaXiabikdaYaqaaiabdEeahbaakiabcYcaSaqaaiabdwha1naaBaaaleaacqaIXaqmcqaI5aqoaeqaaOGaeyypa0JaemiEaG3aa0baaSqaaiabigdaXiabikdaYaqaaiabdsfaubaakiabcYcaSaqaaiabdwha1naaBaaaleaacqaIYaGmcqaIWaamaeqaaOGaeyypa0JaemyEaK3aa0baaSqaaiabigdaXiabikdaYaqaaiabdsfaubaakiabcYcaSaqaaiabdwha1naaBaaaleaacqaIYaGmcqaIXaqmaeqaaOGaeyypa0JaemOEaO3aa0baaSqaaiabigdaXiabikdaYaqaaiabdsfaubaakiabcYcaSaaaaiaawUhaaiaaxMaacaWLjaacbeGae8xnauJae83jaCcaaa@AA82@

{u22=x23A,u23=y23A,u24=z23A,u25=x23C,u26=y23C,u27=z23C,u28=x23G,u29=y23G,u30=z23G,u31=x23T,u32=y23T,u33=z23T.     5''
 MathType@MTEF@5@5@+=feaafiart1ev1aaatCvAUfKttLearuWrP9MDH5MBPbIqV92AaeXatLxBI9gBaebbnrfifHhDYfgasaacH8akY=wiFfYdH8Gipec8Eeeu0xXdbba9frFj0=OqFfea0dXdd9vqai=hGuQ8kuc9pgc9s8qqaq=dirpe0xb9q8qiLsFr0=vr0=vr0dc8meaabaqaciaacaGaaeqabaqabeGadaaakeaadaGabaqaauaabaqaemaaaaqaaiabdwha1naaBaaaleaacqaIYaGmcqaIYaGmaeqaaOGaeyypa0JaemiEaG3aa0baaSqaaiabikdaYiabiodaZaqaaiabdgeabbaakiabcYcaSaqaaiabdwha1naaBaaaleaacqaIYaGmcqaIZaWmaeqaaOGaeyypa0JaemyEaK3aa0baaSqaaiabikdaYiabiodaZaqaaiabdgeabbaakiabcYcaSaqaaiabdwha1naaBaaaleaacqaIYaGmcqaI0aanaeqaaOGaeyypa0JaemOEaO3aa0baaSqaaiabikdaYiabiodaZaqaaiabdgeabbaakiabcYcaSaqaaiabdwha1naaBaaaleaacqaIYaGmcqaI1aqnaeqaaOGaeyypa0JaemiEaG3aa0baaSqaaiabikdaYiabiodaZaqaaiabdoeadbaakiabcYcaSaqaaiabdwha1naaBaaaleaacqaIYaGmcqaI2aGnaeqaaOGaeyypa0JaemyEaK3aa0baaSqaaiabikdaYiabiodaZaqaaiabdoeadbaakiabcYcaSaqaaiabdwha1naaBaaaleaacqaIYaGmcqaI3aWnaeqaaOGaeyypa0JaemOEaO3aa0baaSqaaiabikdaYiabiodaZaqaaiabdoeadbaakiabcYcaSaqaaiabdwha1naaBaaaleaacqaIYaGmcqaI4aaoaeqaaOGaeyypa0JaemiEaG3aa0baaSqaaiabikdaYiabiodaZaqaaiabdEeahbaakiabcYcaSaqaaiabdwha1naaBaaaleaacqaIYaGmcqaI5aqoaeqaaOGaeyypa0JaemyEaK3aa0baaSqaaiabikdaYiabiodaZaqaaiabdEeahbaakiabcYcaSaqaaiabdwha1naaBaaaleaacqaIZaWmcqaIWaamaeqaaOGaeyypa0JaemOEaO3aa0baaSqaaiabikdaYiabiodaZaqaaiabdEeahbaakiabcYcaSaqaaiabdwha1naaBaaaleaacqaIZaWmcqaIXaqmaeqaaOGaeyypa0JaemiEaG3aa0baaSqaaiabikdaYiabiodaZaqaaiabdsfaubaakiabcYcaSaqaaiabdwha1naaBaaaleaacqaIZaWmcqaIYaGmaeqaaOGaeyypa0JaemyEaK3aa0baaSqaaiabikdaYiabiodaZaqaaiabdsfaubaakiabcYcaSaqaaiabdwha1naaBaaaleaacqaIZaWmcqaIZaWmaeqaaOGaeyypa0JaemOEaO3aa0baaSqaaiabikdaYiabiodaZaqaaiabdsfaubaakiabc6caUaaaaiaawUhaaiaaxMaacaWLjaacbeGae8xnauJae83jaCIae83jaCcaaa@ABAD@

Therefore, an ORF or a fragment of DNA sequence can be represented by a point or a vector in the 33-dimensional space V. Note that *u*_*i *_∊ [-1,+1], *i *= 1, 2, ..., 33. Therefore, the space V is a 33-dimensional super-cube with the side length of 2. A total of 33 parameters denoted by u1o−u33o
 MathType@MTEF@5@5@+=feaafiart1ev1aaatCvAUfKttLearuWrP9MDH5MBPbIqV92AaeXatLxBI9gBaebbnrfifHhDYfgasaacH8akY=wiFfYdH8Gipec8Eeeu0xXdbba9frFj0=OqFfea0dXdd9vqai=hGuQ8kuc9pgc9s8qqaq=dirpe0xb9q8qiLsFr0=vr0=vr0dc8meaabaqaciaacaGaaeqabaqabeGadaaakeaaieWacqWF1bqDdaWgaaWcbaGaeGymaeZaaSbaaWqaaiabb+gaVbqabaaaleqaaOGaeyOeI0Iae8xDau3aaSbaaSqaaiabiodaZiabiodaZmaaBaaameaacqqGVbWBaeqaaaWcbeaaaaa@36F7@ are calculated according to the equation (5) for the seed ORF, which corresponds to a point O in the 33-dimensional space. These 33 parameters will be used to differentiate coding/non-coding ORFs.

### (3) Seeking all ORFs and predicting possible protein-coding genes

All the ORFs longer than a given value, for example 90 bp, are extracted as candidates of genes. For each ORF, which is represented by a point in the 33-dimensional space, the Euclidean distance of this point to the point O is obtained

D(u)={∑i=133(ui−uio)2}1/2.     6
 MathType@MTEF@5@5@+=feaafiart1ev1aaatCvAUfKttLearuWrP9MDH5MBPbIqV92AaeXatLxBI9gBaebbnrfifHhDYfgasaacH8akY=wiFfYdH8Gipec8Eeeu0xXdbba9frFj0=OqFfea0dXdd9vqai=hGuQ8kuc9pgc9s8qqaq=dirpe0xb9q8qiLsFr0=vr0=vr0dc8meaabaqaciaacaGaaeqabaqabeGadaaakeaacqWGebarcqGGOaakcqWG1bqDcqGGPaqkcqGH9aqpdaGadaqaamaaqahabaGaeiikaGIaemyDau3aaSbaaSqaaiabbMgaPbqabaGccqGHsislcqWG1bqDdaWgaaWcbaGaeeyAaKMaee4Ba8gabeaakiabcMcaPmaaCaaaleqabaGaeGOmaidaaaqaaiabdMgaPjabg2da9iabigdaXaqaaiabiodaZiabiodaZaqdcqGHris5aaGccaGL7bGaayzFaaWaaWbaaSqabeaacqaIXaqmcqGGVaWlcqaIYaGmaaGccqGGUaGlcaWLjaGaaCzcaGqabiab=zda2aaa@4CDC@

A coding potential index VZ is defined as

VZ=⌊D02−D(u)2⌋/(2×D02)     7
 MathType@MTEF@5@5@+=feaafiart1ev1aaatCvAUfKttLearuWrP9MDH5MBPbIqV92AaeXatLxBI9gBaebbnrfifHhDYfgasaacH8akY=wiFfYdH8Gipec8Eeeu0xXdbba9frFj0=OqFfea0dXdd9vqai=hGuQ8kuc9pgc9s8qqaq=dirpe0xb9q8qiLsFr0=vr0=vr0dc8meaabaqaciaacaGaaeqabaqabeGadaaakeaacqqGwbGvcqqGAbGwcqGH9aqpdaGbdaqaaiabdseaenaaDaaaleaacqaIWaamaeaacqaIYaGmaaGccqGHsislcqWGebarcqGGOaakcqWG1bqDcqGGPaqkdaahaaWcbeqaaiabikdaYaaaaOGaayj84laawUp+aiabc+caViabcIcaOiabikdaYiabgEna0kabdseaenaaDaaaleaacqaIWaamaeaacqaIYaGmaaGccqGGPaqkcaWLjaGaaCzcaGqabiab=Dda3aaa@49B6@

where *D*_0 _is a constant called maximum Euclidean distance, whose default value is 6.90
 MathType@MTEF@5@5@+=feaafiart1ev1aaatCvAUfKttLearuWrP9MDH5MBPbIqV92AaeXatLxBI9gBaebbnrfifHhDYfgasaacH8akY=wiFfYdH8Gipec8Eeeu0xXdbba9frFj0=OqFfea0dXdd9vqai=hGuQ8kuc9pgc9s8qqaq=dirpe0xb9q8qiLsFr0=vr0=vr0dc8meaabaqaciaacaGaaeqabaqabeGadaaakeaadaGcaaqaaiabiAda2iabc6caUiabiMda5iabicdaWaWcbeaaaaa@3093@. All ORFs with VZ scores greater than 0 are regarded as possible protein-coding genes, whereas those with VZ scores less than 0 are regarded as non-coding.

### (4) Dealing with overlapping ORFs

Among all the ORFs having VZ score larger than 0, some ORFs are falsely predicted as genes owing to their overlapping with coding ORFs. In the development of ZCURVE system, a strategy was proposed to deal with overlapping ORFs [11]. Later, this strategy was adopted again in the ZCURVE_CoV system [8]. Here the same strategy is employed once more, while the related parameters are adjusted because of the change of the definition of coding potential score. Briefly, if the VZ score of the longer ORF between the two overlapping ORFs minus a given value is still larger than that of the shorter one, it is recognized as gene, and the shorter is a non-coding one. Otherwise, both are kept as coding. For more detail, refer to [4].

There are three main different features between the present viral gene-finding system ZCURVE_V and our previously reported bacterial gene-finding system ZCURVE. Firstly, two different methods are used to generate seed ORFs: one simply selecting the 'Maximum ORF' and another selecting those long and non-overlapping ORFs as seed ORFs. Secondly, no negative samples (non-coding sequences) are required in the training set of the algorithm for ZCURVE_V system. Thirdly, instead of Fisher linear discriminant algorithm, Euclidean distance discriminant method is used here. Due to the adaptation, the ZCURVE_V system is capable of recognizing protein-coding genes in any anonymous viral or phage genomes, even for those shorter than 1000 bp.

## Availability and requirements

A web interface of the ZCURVE_V system, has been constructed at the site: . When a user pastes a viral or phage genomic sequence into the input window of the homepage, the gene-finding results will be returned to the user immediately. When running ZCURVE_V, a total of 9 specific options are selectable. These options include 'the minimum gene length', 'the maximum Euclidean distance *D*_*0*_', 'the minimum coding potential score VZ', 'belonging to mycoplasma or not', 'being single-stranded DNA/RNA or not', 'the type of start codons', 'keeping overlapping genes or not', 'providing personal seed ORF sequence or not' and 'relocating translation start sites for predicted genes or not', respectively. Registered users may also download the executable version of the program ZCURVE_V, and run it on his (her) computer under the platforms of either Windows (95/98/NT/Me/2000 or higher), or Linux (Redhat 9.0 or higher), or SGI IRIX 6.5. The predicted results for 979 viral and 212 phage genomes are provided through the database named DOVGZ (**D**atabase **O**f **V**iral **G**enes predicted by **Z**CURVE_V), which is available online [24].

## Authors' contributions

CTZ guided the whole study and took part in writing the manuscript. FBG designed the algorithm and wrote the computer program. He also run the program for about 1000 genomes and took part in writing the manuscript.
